# Expanding the watch list for potential Ebola virus antibody escape mutations

**DOI:** 10.1371/journal.pone.0211093

**Published:** 2019-03-21

**Authors:** Jagdish Suresh Patel, Caleb J. Quates, Erin L. Johnson, F. Marty Ytreberg

**Affiliations:** 1 Center for Modeling Complex Interactions, University of Idaho, Moscow, Idaho, United States of America; 2 Department of Biological Sciences, University of Idaho, Moscow, Idaho, United States of America; 3 Department of Physics, University of Idaho, Moscow, Idaho, United States of America; 4 Institute for Bioinformatics and Evolutionary Biology, University of Idaho, Moscow, Idaho, United States of America; Division of Clinical Research, UNITED STATES

## Abstract

The 2014 outbreak of Ebola virus disease (EVD) in Western Africa is the largest recorded filovirus disease outbreak and led to the death of over 11,000 people. The recent EVD outbreaks (since May 2018) in the Democratic Republic of the Congo has already claimed the lives of over 250 people. Tackling Ebola virus (EBOV) outbreaks remains a challenge. Over the years, significant efforts have been put into developing vaccines or antibody therapies which rely on an envelope glycoprotein (GP) of *Zaire ebolavirus* (strain Mayinga-76). Therefore, one key approach for combating EVD epidemics is to predict mutations that may diminish the effectiveness of the treatment. In a previous study we generated a watch list of potential antibody escape mutations of EBOV GP against the monoclonal antibody KZ52. Molecular modeling methods were applied to the three-dimensional experimental structure of EBOV GP bound to KZ52 to predict the effect of every possible single mutation in EBOV GP. The final watch list contained 34 mutations that were predicted to destabilize binding of KZ52 to EBOV GP but did not affect EBOV GP folding and its ability to form trimers. In this study, we expand our watch list by including three more monoclonal antibodies with distinct epitopes on GP, namely Antibody 100 (Ab100), Antibody 114 (Ab114) and 13F6-1-2. Our updated watch list contains 127 mutations, three of which have been seen in humans or are experimentally associated with reduced efficacy of antibody treatment. We believe mutations on this watch list require attention since they provide information about circumstances in which interventions could lose the effectiveness.

## Introduction

*Ebolavirus* has six known species: *Zaire ebolavirus*, *Sudan ebolavirus*, *Taï Forest ebolavirus*, *Bundibugyo ebolavirus*, *Reston ebolavirus* and *Bombali ebolavirus*. [1, 2] Ebola virus (EBOV) is deadly and can led to death in up to 90% cases. The Ebola virus disease (EVD) outbreak in Western Africa between 2014 to 2016 is the largest recorded filovirus disease outbreak and led to death over 11,000 people.[3] This outbreak receded in 2016, but there are two recent EVD outbreak in the Democratic Republic of the Congo that began in May and August 2018 which have already claimed lives of 271 people and more than 458 positive cases (WHO report, 4^th^ December 2018).[4]

The 2014–2016 EVD outbreak in Western Africa promoted discovery of new therapeutics and accelerated development of existing candidates.[5, 6] The success of ZMapp, a cocktail of three chimeric monoclonal antibodies (mAbs) derived from immunized mice, in nonhuman primates (NHP) demonstrated the potential of mAb therapies against EBOV infection, and ZMapp is currently undergoing human trials.[7–9] Subsequently, several other mAbs were isolated or recovered from either experimental animals or human survivors and demonstrated protection against EBOV infection and some of them are currently being used to manage the current EVD outbreak. (https://www.nih.gov/news-events/news-releases/nih-begins-testing-ebola-treatment-early-stage-trial)[10, 11] To prepare for future outbreaks it is critical to anticipate and monitor EBOV evolution since it could lead to antibody escape mutants that could compromise treatment efforts. Sequencing studies conducted on EBOV have revealed a significant genetic variation in EBOV glycoprotein (GP). Sequences recovered during 2014–2016 EVD outbreak in Western Africa have shown 106 out of 676 sites in the GP are affected by the genetic modifications.[12–15] This evolving GP is the target for all the mAbs currently under development and being used in the management of the current EVD outbreak.

To address the potential threat of EBOV evolution outpacing antibody treatment efforts we previously initiated a watch list of potential antibody escape mutants for the EBOV GP.[12] We focused on the KZ52 mAb as it had a 3-D experimental structure bound to EBOV GP available. In this previous study, we combined use of FoldX software with molecular dynamics (MD) simulations to estimate folding and binding stabilities. We placed 34 mutations on the watch list by considering every possible EBOV GP mutation and choosing those that disrupt binding between GP and KZ52 but do not disrupt the ability of GP to fold and bind to form a complex. One of these 34 mutations (*N550K*) was seen in humans in a previous outbreak.[16]

The aim of this study is to expand our watch list of potential antibody escape mutants for EBOV GP. After we published our previous watch list, three more 3-D structures of mAbs interacting with different epitopes on EBOV GP (necessary for our molecular modeling approach) were published in the Protein Data Bank (PDB). In this study, we expand our watch list to include possible antibody escape mutations from three antibodies with distinct epitopes: Antibody 100 (Ab100), Antibody 114 (Ab114) and 13F6-1-2. Our watch list now contains 127 mutations, three of which have been seen in humans (*N550K*)[16] or are experimentally associated with reduced efficacy (*Q406R* and *R409C*)[17] of antibody treatment.

## Methods

For a mutation to be placed on a watch list for EBOV it must: (1) disrupt binding to a protective antibody, and (2) leave the viral proteins functional thus allowing them to fold and assemble. It is thus necessary to determine how amino acid mutations alter stabilities (ΔΔ*G* values) for GP folding, forming a trimer and binding to the antibody. In our previous work, we have obtained ΔΔ*G* values of GP folding, GP trimer formation and GP binding to KZ52 antibody using our molecular dynamics (MD) plus FoldX approach.[12] In this study we calculate ΔΔ*G* values of binding for Ab100, Ab114 and 13F6-1-2 antigen-antibody complexes using the same modeling approach.

### Structure preparation

#### EBOV GP–Ab100 and Ab114 complexes

**Fig 1:**
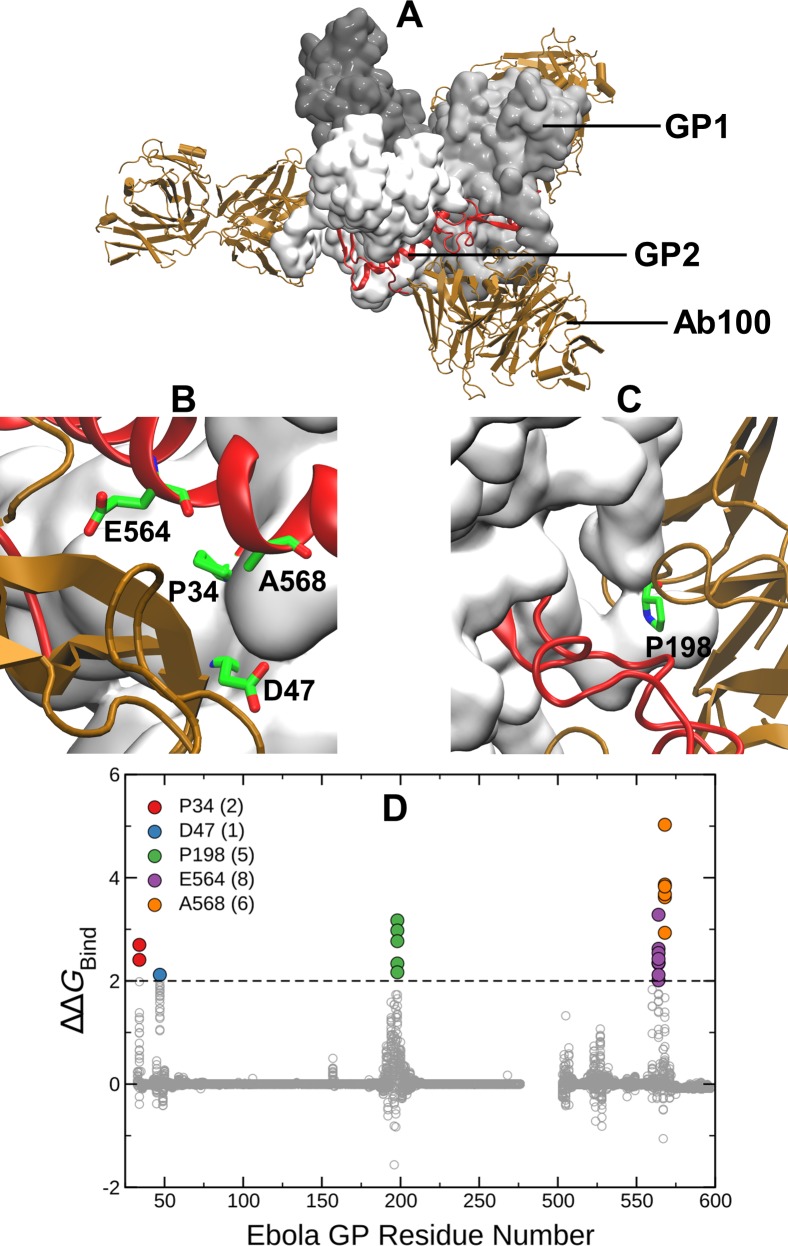
(A) Structure of EBOV GP trimer in complex with the Ab100 antibody. GP1 is gray, GP2 is red and the heavy and the light chains of Ab100 are brown. (B) & (C) Mutations in the GP complex with ΔΔ*G*_Bind_ > 2 kcal/mol (i.e., above black dashed line) are considered disruptive and are highlighted using green stick representation. (D) ΔΔ*G*_Bind_ values (gray circles) for all 19 possible mutations at each site of GP1 (33–278) and GP2 (502–599). Different colors and counts in the legend indicate locations and number of mutations with ΔΔ*G*_Bind_ > 2 kcal/mol on the GP complex.

**Fig 2:**
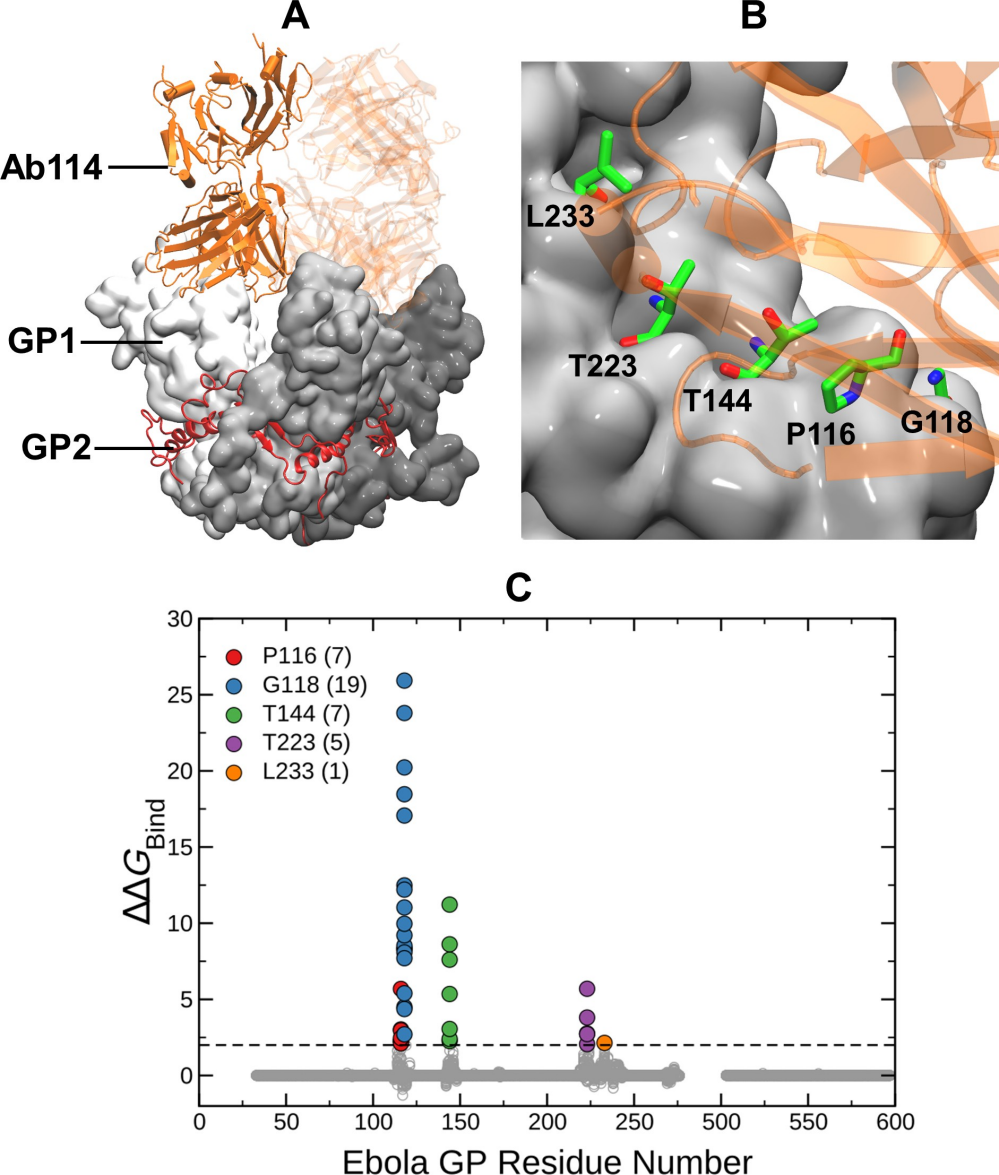
(A) Structure of EBOV GP trimer in complex with the Ab114 antibody. GP1 is gray, GP2 is red and the heavy and the light chains of Ab114 are orange. (B) Mutations in the GP complex with ΔΔ*G*_Bind_ > 2 kcal/mol (i.e., above black dashed line) are considered disruptive and are highlighted using green stick representation. (C) ΔΔ*G*_Bind_ values (gray circles) for all 19 possible mutations at each site of GP1 (33–278) and GP2 (502–599). Different colors and counts in the legend indicate locations and number of mutations with ΔΔ*G*_Bind_ > 2 kcal/mol on the GP complex.

Structures of Ab100 and Ab114 bound to EBOV GP were obtained from the Protein Data Bank (PDB) accession number 5FHC.[18] The EBOV GP amino acid sequence was based on the *Zaire ebolavirus* (strain Mayinga-76), just like our previous study. The PDB file 5fhc.pdb contains coordinates of both Ab100 and Ab114 bound to EBOV GP (GP1 & GP2) monomer at different sites. This file was first modified to remove all but one copy each of GP1, GP2, antibody light chain and antibody heavy chain of Ab100 and Ab114 (one third of the GP-Ab100/Ab114 trimeric complex), and then was split into two files where one file had the GP–Ab100 complex and the other had the GP–Ab114 complex. The MODELLER software[19] was then used to build the missing residues. This included residues 190–213 that are predicted to be intrinsically disordered; the resulting complexes had no secondary structure content in this region. The full EBOV trimer protein complex was then created using the symexp command in PyMOL (see Figs 1A and 2A) and contained three copies each of GP1 (residues 33–278), GP2 (residues 502–599), heavy and light chains of the antibody.

#### EBOV mucin-like domain peptide– 13F6-1-2 antibody complex

**Fig 3:**
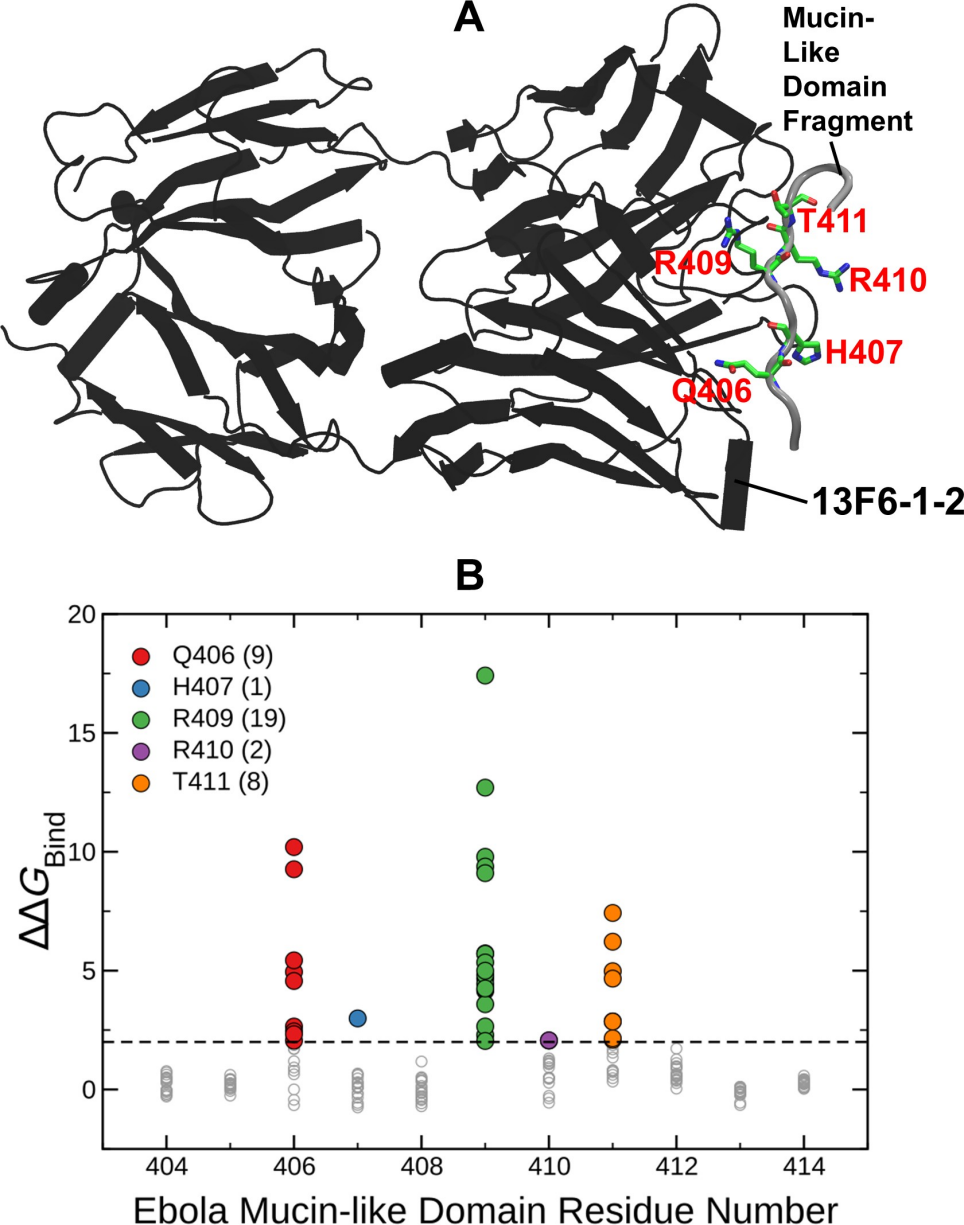
(A) Structure of EBOV GP MLD peptide bound to the 13F6-1-2 antibody. GP MLD peptide is in gray tube representation, and the heavy and the light chains of 13F6-1-2 are black. Mutations in the GP MLD peptide with ΔΔ*G*_Bind_ > 2 kcal/mol (i.e., above black dashed line) are considered disruptive and are highlighted using green stick representation. (B) ΔΔ*G*_Bind_ values (gray circles) for all 19 possible mutations at each of the 11 sites of GP MLD peptide (404–414). Different colors and counts in the legend indicate locations and number of mutations with ΔΔ*G*_Bind_ >2 kcal/mol on the GP complex.

Crystal structure of the 13F6-1-2 Fab fragment bound to its EBOV GP mucin-like domain (GP MLD) peptide epitope (11 amino acids, VEQHHRRTDND) was downloaded from the PDB using the 2QHR accession number.[20] Unlike other epitopes used in our previous and current study, this structure was based on the *Zaire ebolavirus* (strain Eckron-76). However, the alignment of the 11-residue long peptide was 100% identical to the 1976 Mayinga strain. The PDB file 2qhr.pdb was edited to remove everything except GP MLD peptide (residues 404–414) and heavy and light chains of 13F6-1-2 antibody. (see Fig 3A) WHAT IF web server (https://swift.cmbi.umcn.nl) was then used to add the missing atoms to the complex structure.

### Molecular dynamics simulations

All three EBOV–antibody complexes were subjected to atomistic MD simulations using the protocol reported in our previous study.[12] Briefly, the software package GROMACS 5.0.7[21] was used for all MD simulations with the Charmm22* forcefield.[22] GP MLD peptide– 13F6-1-2 simulations were 100 ns, and the GP–Ab100 and Ab114 production simulations were run for a shorter 50 ns due to the large number of atoms in the final simulation box. During the production simulation snapshots were saved every 1 ns resulting in 50 snapshots for each of the GP–Ab100 and GP–Ab114 systems, and 100 snapshots for the peptide– 13F6-1-2 complex.

### FoldX

Snapshots of all three complexes were analyzed using FoldX software.[23, 24] As with our previous study, we began by executing RepairPDB command on each snapshot six times in succession to minimize and obtain convergence of the potential energy. BuildModel command was then used to generate all possible 19 single mutations in EBOV GP and GP MLD peptide at each amino acid site. Lastly, the binding stability of the protein complex due to each mutation was estimated using AnalyseComplex command. For each mutation, we then estimated ΔΔ*G*_Bind_ by averaging the FoldX results across all individual snapshot estimates.

To calculate ΔΔ*G*_Bind_ values for all possible 19 mutations at amino acid site of EBOV GP and GP MLD peptide, we carried out 980,400 FoldX calculations (344 GP residues × 19 possible mutations at each site × 50 MD snapshots × 3 copies of GP-Antibody in a complex) for each GP–Ab100 and GP–Ab114 complexes and 20,900 calculations (11-residue MLD peptide × 19 possible mutations × 100 MD snapshots) for GP MLD peptide– 13F6-1-2 antibody complex. Averaging estimates across all individual snapshots ultimately resulted in 6,441 ΔΔ*G*_Bind_ values each for Ab100 and Ab114 antibody complexes and 209 ΔΔ*G*_Bind_ values for 13F6-1-2 antibody complex. (see S1 File)

## Results and discussion

We have expanded the watch list generated by us in a previous study by including antibody escape mutations against three additional antibodies interacting with EBOV GP. These watch list mutations are those predicted to both disrupt GP—antibody binding and yet allow GP to fold and form trimers.

The EBOV GP is a class I fusion protein consisting of disulfide-linked subunits, GP1 and GP2, that bind to form a chalice-shaped trimer. (see Figs 1A & 2A) Ab100 interacts at the base of the GP trimer (see Fig 1A). This interaction is similar to that of KZ52, the prototypic neutralizing antibody used in our previous study. However, Ab100 contacts GP1 and GP2 of a monomer and the disordered (residue 190–213) loop of the neighboring monomer (see Fig 1A) in contrast to KZ52 which interacts with GP2 of a single monomer. The epitope for Ab114 spans the inner chalice of GP and is in close proximity to the glycan cap, (see Fig 2A) where it remains bound after proteolytic cleavage of the glycan cap and prevents interaction of cleaved GP to its host receptor.[18] 13F6-1-2 is a monoclonal antibody that binds to amino acid residues 405 to 413. This 11-residue peptide shown in the crystal structure is located in the heavily glycosylated mucin-like domain (MLD) of the EBOV GP.[20] (see Fig 3A)

**Fig 4:**
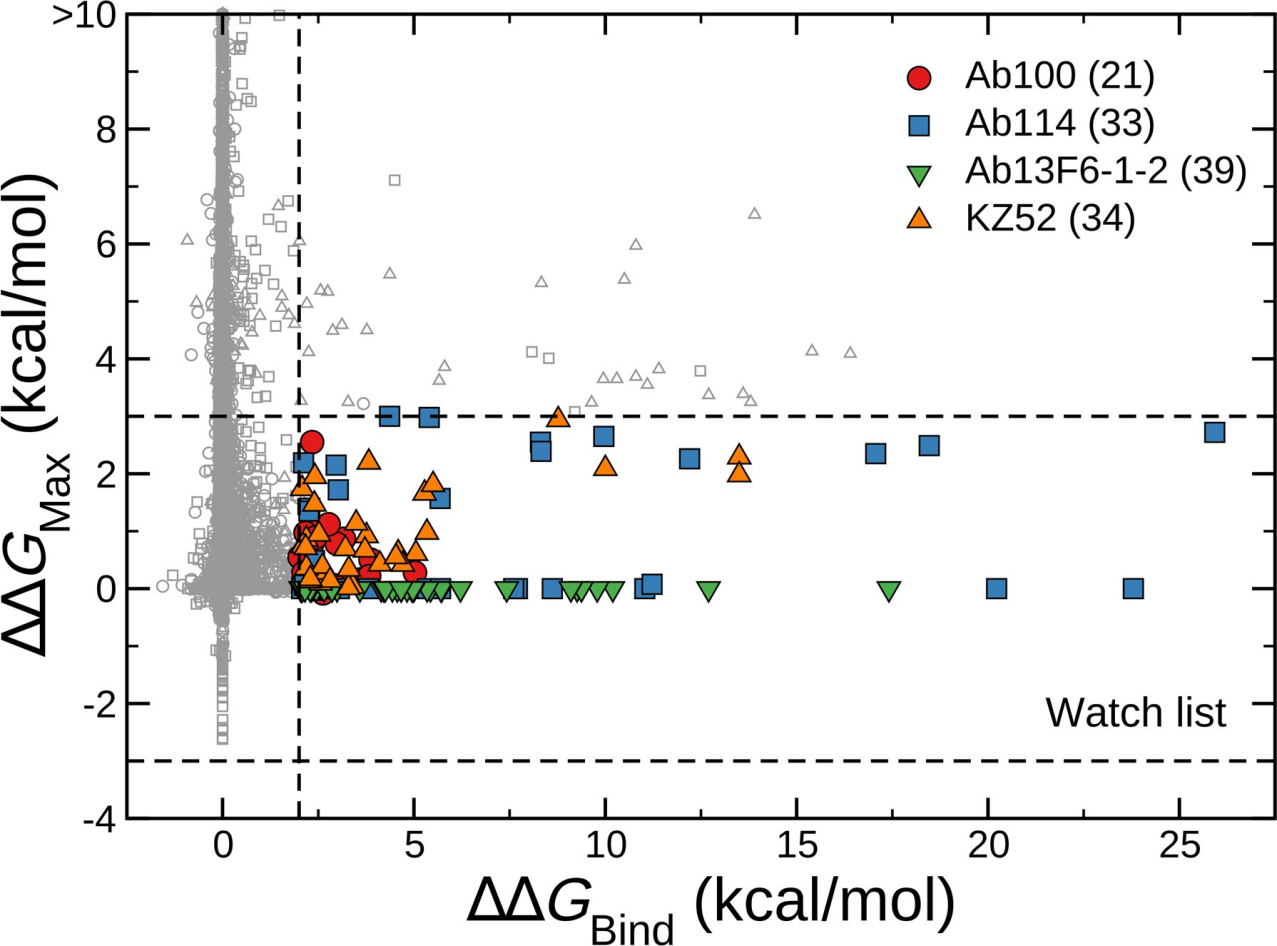
Maximum of folding stability, dimer binding stability (binding of GP1 and GP2) or trimer binding stability (binding of three GP1-GP2 complexes into a trimer of dimers), ΔΔ*G*_Max_, as a function of ΔΔ*G*_Bind_ for all antibody complexes. ΔΔ*G*_Max_ values are considered to be zero for the intrinsically disordered 11-residue MLD peptide. Symbols in the inset legend indicate the corresponding antibody. Watch list mutations are shown as colored symbols and are predicted to disrupt binding to any one of the four antibodies (KZ52, Ab100, Ab114 and 13F6-1-2) but not to disrupt GP folding and trimer formation. Consistent with our previous study, mutations with ΔΔ*G*_Bind_ > 2 kcal/mol are considered disruptive to antibody binding and those with −3 < ΔΔ*G*_Max_ < 3 kcal/mol are considered functional. The number of watch list mutations associated with each antibody is shown in the legend.

Figs 1D, 2C and 3B show that there are 22, 39, 39 mutations respectively against Ab100, Ab114 and 13F6-1-2 predicted to disrupt the binding (ΔΔ*G*_Bind_ > 2 kcal/mol). Fig 4 represents our expanded watch list of EBOV GP mutations against KZ52, Ab100, Ab114 and 13F6-1-2 antibodies. ΔΔ*G*_Max_ is the maximum of folding stability, dimer binding stability (interaction of GP1 and GP2) or trimer binding stability (interaction of a GP1-GP2 dimer with other dimers) and is plotted against the corresponding ΔΔ*G*_Bind_ values for all antibody complexes. The 127 mutations highlighted in colors are part of the watch list as they are predicted to destabilize the antibody binding yet allow EBOV GP to remain functional. There were 21, 33, 39 and 34 watch list mutations respectively in Ab100, Ab114, 13F6-1-2 and KZ52 antibody complexes. Each watch list mutation is given in the Table 1 and shows that they are concentrated at just six residues in KZ52 and at five residues in each of the other three antibody complexes. (see Fig 1B & 1C, Fig 2B and Fig 3A) All of these 21 amino acid sites on EBOV GP are present at the binding interface with mAbs. Interestingly, amino acid changes to Tryptophan (W), Tyrosine (Y), Phenyl alanine (F) and Argenine (R) were seen on 17, 15, 12 and 11 sites respectively out of 21 sites on the watchlist. This clearly suggests that amino acid substitution with bulky side chain at the binding interface is disruptive to the antibody binding.

**Table 1:** Watch list mutations.

Systems	Amino acid site	Predicted Escape Mutations
EBOV GP–KZ52	N506	W^3^, Y^1^
	P513	H^2^, W^3^
	N550	Q^3^, K^3^, P^2^, F^2^, H^1^, I^2^, E^3^, R^2^, W^3^, V^2^, Y^1^, M^3^
	D552	S^2^, Q^3^, K^3^, T^2^, F^2^, A^2^, H^1^, G^2^, R^2^, W^3^, V^2^, Y^1^
	G553	M^3^
	G557	F^3^, H^3^, R^1^, W^1^, Y^3^
EBOV GP–Ab100	P34	W^3^, Y^3^
	D47	R^2^
	P198	F^2^, W^3^, Y^2^, D^2^, H^2^
	E564	K^1^, P^2^, S^2^, T^2^, Y^3^, A^2^, R^2^, G^2^
	A568	F^2^, W^3^, Y^2^, R^2^, H^2^
EBOV GP–Ab114	P116	F^2^, W^3^, Y^2^, R^2^, D^2^, G^2^, H^2^
	G118	L^2^, K^2^, M^2^, F^3^, S^2^, W^1^, Y^3^, R^1^, N^3^, C^3^, Q^2^, E^2^, H^3^
	T144	F^3^, P^1^, W^2^, Y^3^, D^3^, E^2^, H^3^
	T223	F^2^, W^3^, Y^2^, R^2^, H^2^
	L233	W^2^
GP MLD peptide– 13F6-1-2	Q406	R^2^, D^3^, G^2^, H^3^, F^3^, S^2^, T^2^, W^3^, Y^3^
	H407	P^2^
	R409	A^2^, N^2^, D^2^, C^1^, Q^3^, E^3^, G^1^, H^2^, I^2^, L^2^, K^3^, M^3^, F^2^, P^2^, S^1^, T^2^, W^3^, Y^2^, V^2^
	R410	G^1^, W^3^
	T411	R^2^, Q^2^, G^2^, H^3^, K^2^, F^3^, W^3^, Y^3^

Watch list mutations are shown for all four antibodies (KZ52, Ab100, Ab114 and 13F6-1-2) at a predicted amino acid sites on EBOV GP. (See SI S4 File for watch list sorted by amino acid sites) Number in the superscript associated with a watch list mutation denotes a number of nucleotide point mutations required in the genetic code of GP of *Zaire ebolavirus* (strain Mayinga-76; GenBank accession number AF086833) to observe that mutation.

Watch list mutations in Table 1 may act as a reference for public health monitoring agencies. Appearance of any of these mutations in a real population suggests possible diminished effectiveness of these antibodies. One watch list mutation N550K mutation was detected during an EVD outbreak in humans who are believed to have acquired EBOV from gorillas in Central Africa between 2001 and 2003. Moreover, sequencing study carried out on all the isolates from this outbreak revealed the presence of this mutation. N550K has appeared on our watch list suggesting the reduced efficacy of KZ52.[16].

As reported in our previous work,[12] we were able to identify three amino acid sites (N550, D552 and G553; see Table 1) out of five sites (C511, N550, D552, G553 and C556), that were found to be critical in binding of KZ52 to GP in an alanine scanning mutagenesis conducted by Davidson et al.[25] 74% of the watch list mutations predicted against KZ52 occurred at these three sites. (see Table 1) If we ignore our threshold for folding and dimer or trimer formation, we would predict all five crucial sites reported by Davidson et al.[25] Our modeling approach predicted a destabilizing (ΔΔ*G*_Bind_ > 2 kcal/mol) effect for the D552A and G553A mutations but did not show such effect of alanine substitutions at site C511, N550 and C556.[12] It is likely due to the limitation of the FoldX software or possible changes in the conformation caused by the alanine substitution.

In the study of MB-003,[17] a plant-derived monoclonal antibody cocktail composed of c13C6, 13F6-1-2, and c6D8 used effectively in treatment of EBOV virus infection in non-human primates. This cocktail was unable to protect two of six animals when initiated one- or two-days post-infection. Investigation of a mechanism of viral escape in one of the animals showed five nonsynonymous mutations in the monoclonal antibody target sites. Among these mutations *Q406R* and *R409C* were linked to a reduction in 13F6-1-2 antibody binding.[17] Both of these mutations were correctly identified by our modeling strategy and are present on the watch list.

There is evidence to suggest that our watch list may also be applicable to the secreted glycoprotein (sGP). The main protein expressed from *GP* gene is sGP, not the EBOV envelope GP. sGP is dimeric and has a same sequence for first 295 amino acids at N-terminal as envelope GP but has a differing C-terminal region. A study by Iwasa et al.[26] reported that sGP was able to form a complex with GP2 and mimic the role of GP1 in an in vitro analysis. Moreover, they showed that a complex formed by sGP and GP2 was able to interact with KZ52 antibody.

The current expanded watch list includes only four epitopes for which experimental structures of antibodies interacting with viral proteins are available. However, there are other epitopes known for EBOV GP.[10] The availability of more experimental three-dimensional structures would allow the current watch list to grow. In our previous study,[12] we chose a conservative functional zone of −3 < ΔΔ*G*_Max_ < 3 kcal/mol for mutations based on the in-silico stability predictions of the observed functional mutations in a viral coat protein[27] and of 41 mutations detected in 963 EBOV GP sequences. Moreover, our choice of ΔΔ*G*_Bind_ > 2 kcal/mol to define disruption of antibody binding was made by refining the initial threshold to be more inclusive. Further in-depth justification on thresholds is provided in our previous study.[12] However, experimental evaluation is necessary to define these thresholds. The size of our watch list would naturally change with modified thresholds so we have provided spreadsheets with raw ΔΔ*G*_Bind_ data to enable others to build custom watch lists. Our modeling approach predicts ΔΔ*G* value for single amino acid substitutions since it uses FoldX and thus is not appropriate for cases where the antibody binding is disrupted by multiple substitutions. This is because the semi-empirical energy function built into the FoldX software is trained using experimental ΔΔ*G* values of single mutations.[24] Using FoldX to predict ΔΔ*G* values for multiple substitutions is thus expected to yield erroneous results. Moreover, FoldX uses a single three-dimensional static protein structure as an input for predicting ΔΔ*G* values. Therefore, it is unlikely to identify escape mutations occurring at a region other than binding interface or where mutations disrupt binding by altering the conformation of the protein. In vitro validation of the expanded watch list and the accuracy of this approach in predicting escape mutation has not yet been carried out and we hope our study will encourage such research.

In summary, we have expanded the watch list of potential antibody escape mutations of EBOV by including three more antibody complexes: EBOV GP–Ab100, GP–Ab114 and GP Mucin-like domain (GP MLD) peptide– 13F6-1-2. The watch list now contains 127 mutations in 21 sites in EBOV GP. Mutations from the watch list that appear during an outbreak deserve attention since they may be a signal of an evolution of the virus or evolutionary response against the antibody treatment that could reduce the efficacy of treatment efforts. Ab114 has been recently approved for the first time to treat infected individuals during the current EVD outbreak in Democratic Republic of the Congo. (https://www.nih.gov/news-events/news-releases/nih-begins-testing-ebola-treatment-early-stage-trial) We hope our watch list will serve as a useful reference for the public health and emerging infectious disease monitoring agencies. The watch list can still be expanded if more experimental structures of EBOV–antibody complexes become available. In fact, as we were preparing this manuscript, the crystal structure of mAb CA45 bound to GP1 and GP2 interface of EBOV GP were published by Janus et al.[28] Lastly, we believe our in-silico approach could be applied to determine watch lists for other viruses provided experimental structures are available and for future design and optimization efforts of antibodies.

## Supporting information

S1 File Excel spreadsheet with estimated stability effects of all 6,441 mutations against Ab100. (XLSX)

S2 File Excel spreadsheet with estimated stability effects of all 6,441 mutations against Ab114. (XLSX)

S3 File Excel spreadsheet with estimated stability effects of all 209 mutations against 13F6-1-2. (XLSX)

S4 File Excel spreadsheet with estimated binding stability effects of watch list mutations against all the four antibodies (KZ52, Ab100, Ab114, and 13F6-1-2) and a number of nucleotide point mutations required in the genetic code of GP of *Zaire ebolavirus* (strain Mayinga-76; GenBank accession number AF086833) to observe each mutation. (XLSX)
